# Understanding the characteristics of high users of hospital services in Singapore and their associations with healthcare utilisation and mortality: A cluster analysis

**DOI:** 10.1371/journal.pone.0288441

**Published:** 2023-07-11

**Authors:** Mimaika Luluina Ginting, Yan Hoon Ang, Soon Hoe Ho, Grace Sum, Chek Hooi Wong

**Affiliations:** 1 Geriatric Education and Research Institute Singapore, Singapore, Singapore; 2 Department of Geriatric Medicine, Khoo Teck Puat Hospital, Singapore, Singapore; 3 Health Services & Systems Research, Duke-NUS, Singapore, Singapore; TERI School of Advanced Studies, INDIA

## Abstract

**Introduction:**

High users of hospital services require targeted healthcare services planning for effective resource allocation due to their high costs. This study aims to segmentize the population in the “Ageing In Place-Community Care Team” (AIP-CCT), a programme for complex patients with high inpatient service use, and examine the association of segment membership and healthcare utilisation and mortality.

**Methods:**

We analysed 1,012 patients enrolled between June 2016 and February 2017. To identify patient segments, a cluster analysis was performed based on medical complexity and psychosocial needs. Next, multivariable negative binomial regression was performed using patient segments as the predictor, with healthcare and programme utilisation over the 180-day follow-up as outcomes. Multivariate cox proportional hazard regression was applied to assess the time to first hospital admission and mortality between segments within the 180-day follow-up. All models were adjusted for age, gender, ethnicity, ward class, and baseline healthcare utilisation.

**Results:**

Three distinct segments were identified (Segment 1 (n = 236), Segment 2 (n = 331), and Segment 3 (n = 445)). Medical, functional, and psychosocial needs of individuals were significantly different between segments (*p*-value<0.001). The rates of hospitalisation in Segments 1 (IRR = 1.63, 95%CI:1.3–2.1) and 2 (IRR = 2.11, 95%CI:1.7–2.6) were significantly higher than in Segment 3 on follow-up. Similarly, both Segments 1 (IRR = 1.76, 95%CI:1.6–2.0) and 2 (IRR = 1.25, 95%CI:1.1–1.4) had higher rates of programme utilisation compared to Segment 3. Patients in Segments 1 (HR = 2.48, 95%CI:1.5–4.1) and 2 (HR = 2.25, 95%CI:1.3–3.6) also had higher mortality on follow-up.

**Conclusions:**

This study provided a data-based approach to understanding healthcare needs among complex patients with high inpatient services utilisation. Resources and interventions can be tailored according to the differences in needs among segments, to facilitate better allocation.

## Introduction

Globally, there is an increasing trend of population ageing with multiple chronic conditions, mental health and medication-related problems, and social vulnerability. This has contributed to a shift towards more complex needs of the aging population [1]. Healthcare delivery systems and organisations that were initially developed in response to acute needs have been facing challenges in integrating care to address the complex needs of patients [2]. Older adults are often high users of inpatient services with repeated hospitalisations, and increasing challenges in care transition have resulted in hospital readmission, mortality, and higher healthcare cost [3]. Previous studies reported high healthcare costs associated with multiple comorbidities, mental health problems, increasing age, and end-of-life care [4–6].

The strain on healthcare resources has increased calls for more efficient and improved strategies in healthcare services, as the Organization for Economic Cooperation and Development reported high users of hospital resources with estimates ranging from 10% to 34% of healthcare expenditures [7]. Optimal management with a more targeted, efficient, and coordinated healthcare services by having a more person-centered approach in understanding these “high user” patients should be sought to facilitate better care to address their complex needs and to facilitate better resource allocation [8].

While it is impossible to develop care models for every individual at a population level, understanding the needs around groups of people with similar characteristics could aid in resource allocation and programme planning. Identifying these groups, known as population segments, can allow optimisation of healthcare service planning at population level and develop integrated healthcare system that is more targeted and efficient [9–11].

Population segmentation uses variables or characteristics used to assign population to homogeneous groups at various levels including–macro (e.g., general population), meso (e.g., specific population with certain disease or conditions, i.e., diabetic patients), and micro (e.g., individual adverse event risk stratification) [10,12]. Derivation of segments could be done through expert inputs (a priori) or post-hoc using statistical methods applied to empirical data [13,14]. Most population segmentation studies often use healthcare utilisation, medical, and socio-demographic characteristics as the basis of segmentation and fewer studies used functional and social variables [2,15,16]. There is increasing evidence that using medical complexity alone is insufficient in explaining healthcare utilisation [3]. A more comprehensive look to include other domains of psychosocial needs is also considered important in understanding the factors driving healthcare utilisation and guiding risk stratification and segmentation to improve care delivery for these patients [3].

This study segmentizes a high user patient population in the Ageing In Place-Community Care Team (AIP-CCT) programme, a programme for complex patients with high inpatient service use, into segments with distinct characteristics, based on their medical complexity and psychosocial needs. We defined high users as complex patients with high inpatient service usage, specifically, those with three or more hospital admissions in the past 12 months since the enrolment in AIP-CCT programme. We also explore the association between segment membership and healthcare utilisation (hospital admissions, length of hospital stay (LOS), emergency department (ED) visits, AIP-CCT programme utilisation) and mortality at 180-day follow-up.

## Materials and methods

### Sample and data source

We used the National Healthcare Group (NHG) Regional Health System (RHS) database. The database included data on socio-demographics, primary and secondary diagnosis (based on International Classification of Diseases 10th Revisions), public healthcare utilisation (hospital admissions, day surgeries, ED visit), hospital length of stay, and mortality. We also used the AIP-CCT programme administrative database, which included data on the programme’s home visit assessment. These include socio-demographic profile, medical, functional and psychosocial assessment, and programme utilisation. We included all patients enrolled into AIP-CCT between June 2016 and February 2017 (n = 1,326). After excluding 312 patients with incomplete data, our study analysed a total of 1,012 AIP-CCT patients. For each patient, the first AIP-CCT home visit was identified as the index time for analysis. The baseline period was defined as the 180 days prior to the index time. The follow-up period was the 180 days immediately following the first home visit assessment.

### Programme description

The AIP-CCT programme was a homecare service that delivers multi-disciplinary care to a high user patient population. This population had complex needs with progressive or life-limiting conditions, with high inpatient services use (Table 1). The high service use refers to three or more hospital admissions in the past 12 months from programme enrolment. The delivery of care was guided by a comprehensive needs assessment of the medical, functional, nursing, and psychosocial profiles of the patient in Singapore Northern region.

**Table 1:** AIP-CCT programme inclusion and exclusion criteria.

Inclusion criteria	Exclusion criteria
Patients to meet at least one of the following: 1. Older adult with dementia, frequent falls, functional decline post-hospitalisation or urinary/bowel incontinence 2. Complex medical conditions such as: • Chronic obstructive pulmonary disease, congestive heart failure or chronic kidney disease with ≥3 admissions in last 1 year • Poorly controlled diabetes mellitus • Acute stroke with new disability • Thromboembolism that requires anticoagulation 3. Complex nursing needs with stage 3 or 4 pressure sores, infected wounds, stoma care or intermittent urinary catheterisation 4. Socially at risk individuals with inadequate care or need caregiver training 5. End of life patients	Discharged to institutional care or other community support services (such as community hospitals, home hospice, inpatient hospice, nursing homes, home medical, home nursing and social day care).

The AIP-CCT is a hospital-led programme which offered home visits and tele-consultations to support patients and caregivers in managing chronic diseases for 3 months after being enrolled into the programme. The programme aimed to address the unmet needs of patients by giving support to the patients/caregivers in managing chronic disease to reduce patients’ acute hospital utilisation. It also provided case management to link patients to community services for their social needs. Potential patients referred from hospital inpatient wards, specialist outpatient clinics (SOC), and the ED were triaged and linked to a community team based on their assessed needs. The team comprised of nurses, doctors, physiotherapists, occupational therapists, speech therapists, pharmacists, medical social workers, and healthcare assistants [17]. If there was continuing needs beyond the 3 months period, patients would be referred to community long term Home Medical and Home Nursing Services ran by Social Service Agencies, which are non-profit organisations that provide welfare services and/or services that benefit the community at large [18].

### Cluster analysis approach

We performed a cluster analysis to generate non-overlapping population segments. Clustering aimed to partition a set of data points into segments (clusters), so that the data points were more similar to each other than data points in different segments. Medical complexity and psychosocial needs were used as input variables to assign observations into homogeneous segments in the clustering process.

Medical complexity was measured using the Charlson Comorbidity Index (CCI). CCI is widely used as a measure of comorbidity, with higher score indicating higher mortality risk and more severe comorbid conditions [19]. It provides a valid assessment of individual’s unique clinical condition [19]. The chronic conditions used to compute CCI included myocardial infarction, congestive heart failure, peripheral vascular disease, cerebrovascular disease, dementia, chronic pulmonary disease, rheumatic disease, mild/moderate/severe liver disease, diabetes with and without chronic complication, hemiplegia or paraplegia, renal disease, any malignancy, metastatic solid tumor, and HIV/AIDS [20].

Psychosocial need was measured using Social Triage (ST) score, which was a composite variable on patient/family social support, patient’s mental health, treatment compliance, and patient/family coping response. Patient/family social support was assessed by examining the availability of existing caregiver or formal/informal social support for patient. Patient’s mental health was assessed by examining the existence of mental condition e.g., dementia, depression, psychiatric/substance abuse, alcoholism, or other behaviour problem that affected care. Treatment compliance was based on patient’s adherence to treatment/care plans. Patient/family coping response was assessed by rating their ability to cope with diseases. Each component of the composite variable has a 3-point rating score, with higher score indicating poorer support, more mental health issues, poor treatment compliance, and poor coping response, respectively. S1 Table describes each component within the ST score.

We conducted the cluster analysis using the Partitioning Around Medoids (PAM) approach. PAM is a simple unsupervised machine learning clustering algorithm that groups data into a specified number (*k*) of clusters (segments). This approach searches for representative observations in the dataset called *medoids* that are centrally located in clusters, and then assigns all other observations to the closest *medoid*, in order to create clusters [21,22].

### Determining the optimal number of segments

We applied a post-hoc approach to define the optimal number of segments presented within the data set, using the R package NbClust [23]. NbClust provides 30 indices which estimate the optimal number of segments in a data set and proposes the best clustering scheme from different results obtained by varying all combinations of number of segments, distance measures, and clustering methods [23]. We selected the optimal number of segments with a majority rule, which is the number of segments derived from majority of the indices. The final segmentation outcome was assessed by its clinical relevance and interpretability, to evaluate the goodness of clustering algorithm results.

### Statistical analyses

Sociodemographic, clinical, functional, and psychosocial variables, and healthcare utilisation pattern were collected at baseline to describe the characteristics of each segment of patients. Chi-square/Fisher exact test and one-way ANOVA/Kruskal-Wallis H tests were used to determine whether there were statistically significant differences in the baseline characteristics across the patient segments for categorical and continuous variables (parametric and non-parametric), respectively.

Next, we examined the association of segment membership and prospective healthcare utilisation and mortality. We performed multivariable negative binomial regression model using the patient segments as the predictor and the number of hospital admissions, LOS, ED visits, and programme utilisation over the 180-day follow-up period as outcomes. In addition, we applied multivariable cox proportional hazard regression model to assess and compare the time to the first hospital admission and mortality between segments within the 180-day follow-up period. All models were adjusted for age, gender, ethnicity, ward class, and baseline healthcare utilisation. Ward class refers to the highest tier of hospital ward where the patient had stayed among his/her all hospital admissions. Different ward classes have different facilities, charges and level of subsidy for hospitalisation costs, but the quality of medical care remains the same in all wards. All analyses were performed in R version 3.6.1 (R Core Team, 2019).

## Results

### Baseline characteristics of the three patient segments

Among all indices within the NbClust package, majority (9 of 30 indices) proposed 3 as the best number of segments. Following the majority rule, we ran the PAM algorithm with *k* = 3 in the study population.

There were a total of 1,012 patients across all three segments. The mean age of patients was 75.8 years (Standard Deviation (SD): 12.5). Majority of the patients were female (55.5%), married (48.2%), and of Chinese ethnicity (61.7%). Majority lived in 4 rooms public housing apartment (43%). Table 2 presents the baseline characteristics of the three patient segments.

**Table 2:** Baseline characteristics of the patient segments.

Variables	Pooled (n = 1012)	Segment 1 (n = 236)	Segment 2 (n = 331)	Segment 3 (n = 445)	*p*-value^a^
***Socio-demographics***					
**Age, mean (SD)**	75.8 (12.5)	76.8 (13.0)	75.9 (11.2)	75.3 (13.2)	0.128
**Gender, n (%)**					0.012
Male	450 (44.5)	111 (47.0)	164 (49.6)	175 (39.3)	
Female	562 (55.5)	125 (53.0)	167 (50.5)	270 (60.7)	
**Ethnicity, n (%)**					0.002
Chinese	623 (61.6)	172 (72.9)	185 (55.9)	266 (59.8)	
Malay	218 (21.5)	36 (15.3)	80 (24.2)	102 (22.9)	
Indian	108 (10.7)	19 (8.1)	37 (11.2)	52 (11.7)	
Others	63 (6.2)	9 (3.8)	29 (8.8)	25 (5.6)	
**Marital status, n (%)**					<0.001
Single	70 (6.92)	32 (13.6)	11 (3.3)	27 (6.1)	
Married	488 (48.2)	102 (43.2)	183 (55.3)	203 (45.6)	
Widowed	425 (42.0)	95 (40.3)	130 (39.3)	200 (44.9)	
Divorced / Separated	29 (2.87)	7 (2.97)	7 (2.1)	15 (3.4)	
**Housing type, n (%)**					
***Smaller housing type***					<0.001
1–2 room public housing apartment	89 (8.8)	36 (15.3)	30 (9.1)	23 (5.2)	
3 rooms public housing apartment	197 (19.5)	64 (27.1)	58 (17.5)	75 (16.9)	
***Larger housing type***					
4 rooms public housing apartment	435 (42.98)	84 (35.6)	139 (41.99)	212 (47.6)	
5 rooms and above public housing apartment	238 (23.5)	41 (17.4)	87 (26.3)	110 (24.7)	
Private housing	53 (5.2)	11 (4.7)	17 (5.1)	25 (5.6)	
**Ward class** ^**b**^ **, n (%)**					<0.001
A Class	25 (2.5)	2 (0.9)	8 (2.4)	15 (3.4)	
B1 Class	33 (3.3)	5 (2.1)	9 (2.7)	19 (4.3)	
B2 Class	471 (46.5)	84 (35.6)	169 (51.1)	218 (48.99)	
C Class	483 (47.7)	145 (61.4)	145 (43.8)	193 (43.4)	
***Medical***					
**Charlson Comorbidity Index**^**c**^ **score, mean (SD)**	2.4 (2.3)	2.3 (2.3)	4.5 (2.0)	0.9 (0.8)	<0.001
***Functional***					
**Barthel Activities of Daily Living score, mean (SD)**	60.9 (36.0)	49.1 (37.4)	60.8 (34.2)	67.3 (34.9)	<0.001
**Barthel Activities of Daily Living category, n (%)**					<0.001
Total dependence	223 (22.0)	83 (35.2)	66 (19.9)	74 (16.6)	
Severe dependence	187 (18.5)	48 (20.3)	69 (20.8)	70 (15.7)	
Moderate dependence	286 (28.3)	52 (22.0)	107 (32.3)	127 (28.5)	
Slight dependence	199 (19.7)	28 (11.9)	62 (18.7)	109 (24.5)	
Independent	117 (11.6)	25 (10.6)	27 (8.2)	65 (14.6)	
**Instrumental Activities of Daily Living score, mean (SD)**	2.6 (2.8)	2.2 (2.8)	2.3 (2.5)	3.1 (2.9)	<0.001
***Psychosocial***					
**Social Triage**^**d**^ **score, mean (SD)**	4.97 (1.5)	7.4 (1.2)	4.3 (0.6)	4.2 (0.4)	<0.001
**Social Triage category** ^**e**^ **, n (%)**					<0.001
Low risk	908 (89.7)	132 (55.9)	331 (100)	445 (100)	
Moderate risk	94 (9.3)	94 (39.8)	0	0	
High risk	10 (1.0)	10 (4.2)	0	0	
**Patient/family social support** ^**f**^ **, n (%)**					<0.001
Poor	39 (3.9)	36 (15.3)	3 (0.9)	0	
Fair	175 (17.3)	138 (58.5)	26 (7.9)	11 (2.5)	
Strong	798 (78.9)	62 (26.3)	302 (91.2)	434 (97.5)	
**Patient’s mental health, n (%)**					<0.001
Has mental health that affect care	21 (2.1)	21 (8.9)	0	0	
Has mental health but not affecting care	243 (24.0)	146 (61.9)	44 (13.3)	53 (11.9)	
Good mental health	748 (73.9)	69 (29.2)	287 (86.7)	392 (88.1)	
**Patient treatment compliance, n (%)**					<0.001
Poor	27 (2.7)	26 (11)	1 (0.3)	0	
Fair	166 (16.4)	142 (60.2)	13 (3.9)	11 (2.5)	
Good	819 (80.9)	68 (28.8)	317 (95.8)	434 (97.5)	
**Patient/family coping response** ^**g**^ **, n (%)**					<0.001
Good	807 (79.7)	57 (24.2)	317 (95.8)	433 (97.3)	
Moderate	182 (18.0)	156 (66.1)	14 (4.2)	12 (2.7)	
Poor	23 (9.7)	23 (9.7)	0	0	
**Baseline healthcare utilisation**					
Number of ED visits at baseline, mean (SD)	2.1 (2.1)	2 (2.1)	2.3 (3.0)	1.9 (1.1)	0.006
Number of hospital admissions at baseline, mean (SD)	1.8 (1.2)	1.7 (1.4)	2.1 (1.2)	1.7 (0.9)	<0.001
Number of SOC visits at baseline, mean (SD)	2.4 (3.0)	2.2 (3.1)	3.1 (3.5)	2.1 (2.5)	<0.001

SD: Standard deviation.

^a^p-values come from the Chi-square/Fisher exact test and one-way ANOVA/Kruskal-Wallis H tests for categorical and continuous variables (parametric and non-parametric), respectively. It reflects the significance difference across segments.

^b^Ward class refers to the highest tier of hospital ward where the patients had stayed among his/her all hospital admissions. A Class is the highest tier ward with single-bed, and not subsidised. B1 Class has 4 beds and a 20% subsidy. B2 Class has 6 beds and a 50–65% subsidy. C Class has 8–10 beds and a 65–80% subsidy for hospitalisation costs.

^c^Charlson Comorbidity Index (CCI) is a proxy for medical complexity and the severity of comorbidity. It predicts the one-year mortality for a patient who may have a range of comorbid conditions.

^d^Social triage is a composite score that assess the individual’s risk of having psychosocial issues needing intervention i.e. referral to medical social worker. It consists of four assessments: (i) the availability of patient’s family/social support, (ii) patient’s mental health, (iii) treatment compliance, and (iv) patient/family coping response. Higher score signifies higher psychosocial needs.

^e^Score-based categorisation: Low risk = 4–7, moderate risk = 8–9, and high risk = 10–12.

^f^Poor = patient without caregiver or with caregiver and unwilling/unable to provide support. Fair = patient with existing caregiver and expresses caregiver burden, but open to options. Strong = patient with existing caregiver/formal or informal social support who is willing to provide care.

^g^Good = family/patient accepts condition and is able to work on issues. Moderate = family/patient accepts condition, is still grieving/anxious but within control. Poor = family/patient is in shock, not accepting condition, grieving intensely/highly anxious.

There were no significant differences in age among segments. The three segments had significantly different medical complexity and psychosocial needs, as reflected by the CCI (*p*-value = <0.001) and ST score (*p*-value = <0.001). This reflects the central aim of cluster analysis which is to maximise the distance between clustering variables. Segment 2 had the highest medical complexity with highest mean CCI score 4.5 (SD: 2.0), followed by Segment 1 mean CCI score 2.3 (SD: 2.3) and Segment 3 mean CCI score 0.9 (SD: 0.8). Segment 1 had the highest psychosocial needs as reflected by the highest mean ST score 7.4 (SD: 1.2) with poorer patient/family social support, mental health, treatment compliance and coping response, followed by Segment 2 (mean ST score 4.3 (SD: 0.6)) and Segment 1 (mean ST score 4.2 (SD: 0.4)).

In addition, the non-clustering variables including functional and baseline healthcare utilisation differed significantly. This demonstrated that each segment was largely distinct. The Barthel Activities of Daily Living (ADL) (*p*-value = <0.001) and Instrumental Activities of Daily Living (IADL) (*p*-value = <0.001) score were significantly different across segments. Segment 1 had the highest functional limitation with the lowest mean ADL score of 49.1 (SD: 37.4) and lowest mean IADL score 2.2 (SD: 2.8), followed by Segment 2 (mean ADL score 60.8 (SD: 34.2) and mean IADL score 2.3 (SD: 2.5)), and Segment 3 (mean ADL score 67.3 (SD: 34.9) and mean IADL score 3.1 (SD: 2.9)). Baseline healthcare utilisation were also significantly different among the three segments. Segment 2 had the highest number of ED visits (mean = 2.3, SD: 3.0), hospital admissions (mean = 2.1, SD: 1.2), and SOC visits (mean = 3.1, SD: 3.5) at baseline, followed by Segment 1 (ED visits (mean = 2, SD: 2.1), hospital admissions (mean = 1.7, SD: 1.4), and SOC visits (mean = 2.2, SD: 3.1)), and Segment 3 (ED visits (mean = 1.9, SD: 1.1), hospital admissions (mean = 1.7, SD: 0.9), and SOC visits (mean = 2.1, SD: 2.5)).

### Healthcare utilisation in the follow-up period

We examined the number of hospital admissions, LOS, ED visits, and SOC visits in the 180-day follow-up period using the multivariable negative binomial model, adjusting for age, gender, ethnicity, ward class, and all baseline healthcare utilisation (i.e., number of ED visits at baseline, number of hospital admissions at baseline, and number of SOC visits at baseline) (Table 3).

**Table 3:** Hospital admission, LOS, ED visit, SOC visit, and Programme utilisation in the 180-day follow-up across patient segments.

	Incidence Rate Ratio	95% Confidence Interval	p-value^a^
**Number of hospital admission during the 180-day follow-up**			
Segment 3	1.00	Reference	
Segment 1	1.63	1.3–2.1	<0.001
Segment 2	2.11	1.7–2.6	<0.001
**Length of hospital stay during the 180-day follow-up**			
Segment 3	1.00	Reference	
Segment 1	2.45	1.7–3.5	<0.001
Segment 2	2.63	1.9–3.7	<0.001
**Number of Emergency Department visit during the 180-day follow-up**			
Segment 3	1.00	Reference	
Segment 1	1.56	1.3–1.9	<0.001
Segment 2	1.86	1.5–2.3	<0.001
**Number of Specialist Outpatient Clinic visit during the 180-day follow-up**			
Segment 3	1.00	Reference	
Segment 1	0.78	0.67–0.9	0.002
Segment 2	0.99	0.86–0.98	0.857
**Number of programme utilisation**^**b**^ **during the 180-day follow-up**			
Segment 3	1.00	Reference	
Segment 1	1.76	1.6–2.0	<0.001
Segment 2	1.25	1.1–1.4	<0.001

^a^Multivariate negative binomial regression models adjusted for age, gender, ethnicity, ward class, and all baseline healthcare utlisation (i.e., number of ED visits at baseline, number of hospital admissions at baseline, and number of SOC visits at baseline).

^b^Programme utilisation included home visits done by either a doctor, nurse, physiotherapist, speech therapist, or occupational therapist.

Compared to those in Segment 3, the rate of getting hospitalised for patients in Segment 2 was approximately two-fold higher (Incidence Rate Ratio (IRR) = 2.11, 95% CI: 1.7 to 2.6) and 1.6 times higher in Segment 1 (IRR = 1.63, 95% CI: 1.3 to 2.1). Compared to Segment 3, patients in Segment 2 had a rate 2.6 times higher for LOS (in days) (IRR = 2.63, 95% CI: 1.9 to 3.65), and those in Segment 1 had a rate 2.5 times higher for LOS (in days) (IRR = 2.45, 95% CI: 1.71 to 3.5). Compared to Segment 3, the rate of having ED visits was greater in Segment 2 (IRR = 1.86, 95% CI: 1.5 to 2.3) and Segment 1 (IRR = 1.56, 95% CI: 1.3 to 1.9). However, compared to Segment 3, the rate of having SOC visits was lower in Segment 1 (IRR = 0.78, 95% CI: 0.67 to 0.9).

### Programme utilisation in the follow-up period

We examined programme utilisation in the 180-day follow-up period using the multivariable negative binomial model adjusting for age, gender, ethnicity, ward class, and baseline healthcare utilisation (Table 3). Compared to Segment 3, the rate of having home visits was 76% more in Segment 1 (IRR = 1.76, 95% CI: 1.6–2.0) and 25% more in Segment 2 (IRR = 1.25, 95% CI: 1.1–1.4).

### Time to first hospital admission and mortality

We examined time to first hospital admission and time to mortality during the 180-day follow-up, using cox proportional hazard regression models adjusting for age, gender, ethnicity, ward class, and all baseline healthcare utilisation (i.e., number of ED visits at baseline, number of hospital admissions at baseline, and number of SOC visits at baseline) (Table 4).

**Table 4:** Time to first hospital admission and mortality during the 180-day follow-up across patient segments.

	Hazard Ratio	95% Confidence Interval	*p*-value^a^
**Time to first hospital admission at 180-day follow-up**			
Segment 3	1.00	Reference	
Segment 1	1.62	1.3–2.1	<0.001
Segment 2	2.13	1.7–2.6	<0.001
**Time to mortality at 180-day follow-up**			
Segment 3	1.00	Reference	
Segment 1	2.48	1.5–4.1	<0.001
Segment 2	2.25	1.4–3.6	0.001

^a^Multivariate negative binomial regression models adjusted for age, gender, ethnicity, ward class, and all baseline healthcare utlisation (i.e., number of ED visits at baseline, number of hospital admissions at baseline, and number of SOC visits at baseline).

Compared to Segment 3, there was a higher risk of having their first hospital admission for those in Segment 2 (Hazard Ratio (HR) = 2.13, 95% CI: 1.7 to 2.6) and Segment 1 (HR = 1.62, 95% CI: 1.3 to 2.1). Compared to Segment 3, there was an approximately two-fold higher risk of dying among those in Segment 1 (HR = 2.48, 95% CI: 1.5 to 4.1) and Segment 2 (HR = 2.25, 95% CI: 1.4 to 3.6).

## Discussion

**Fig 1:**
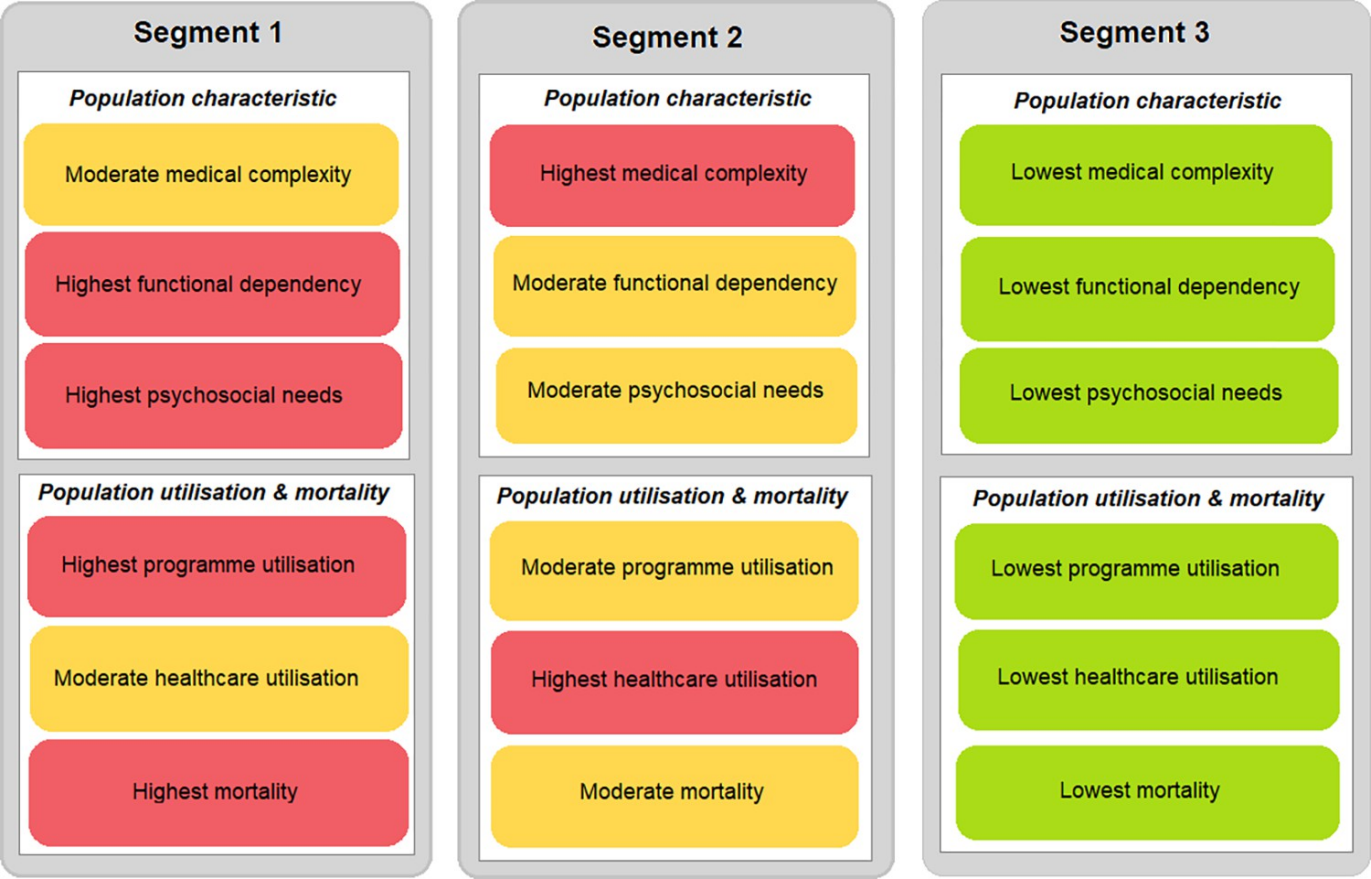
Segment characteristics.

The cluster analysis demonstrated in this study identified three patient segments based on medical complexity and psychosocial needs variables. The three segments had significantly different medical complexity and psychosocial needs, as clustering analysis maximised the distance between the segmentation variables.[24] In addition, other non-clustering variables i.e., ADL, IADL, and baseline healthcare utilisation, were also found to differ significantly, demonstrating that each segment was largely distinct. Fig 1 illustrates the differences among segments.

### Population characteristics

Of the three segments, Segment 1 had the highest functional dependency and psychosocial needs with 35% of the segment having a total dependence based on ADL score category, lowest IADL score and the highest risk of having psychosocial needs based on the ST score. Segment 2 had the highest medical complexity, reflected by the highest CCI score. Segment 3 had the lowest medical complexity, functional dependency and psychosocial needs, hence this segment was used as a reference group for the multivariable analysis.

### Population utilisation and mortality

Our study examined the association between membership across different segments with healthcare utilisation and mortality at 180-day follow-up. We found that the rate of having hospital admission, LOS (in days), ED visit, SOC visit, programme utilisation, and risk of hospital admission and mortality in the follow-up period differed across the three patient segments, suggesting that our segmentation approach could arrive at population segments with varying risk of healthcare utilisation and mortality.

Patients in Segment 1 with the highest functional dependency and psychosocial needs had the highest programme utilisation with moderate rate of hospitalisation and ED visit, and the highest risk of dying in the follow-up period. It is noteworthy that this Segment also had moderate medical complexity, with an average CCI score of 2.3 (SD: 2.3). Another study reported that individuals who were physically dependent had higher odds of mortality in the follow-up period as compared to those who were largely independent, even when both groups had similar level of comorbidity [25]. This suggests that physical dependency might serve as a differentiating factor that contributes to higher odds of mortality in the former group. A study amongst older adults with multimorbidity reported that physical functioning acted as a mediator of the multimorbidity and mortality association [26]. Literature also reported that psychosocial factors, e.g., social support, mental health, coping ability, are important predictors of mortality beyond severity of comorbidity. This might relate to the effect of these factors towards a negative influence in physical health. It is plausible that this might involve mechanisms that induced stress, which may affect health in various ways, such as via endocrine and / or immune system [27].

Patients in Segment 2 with the highest medical complexity reflecting more medical needs had the highest risk of healthcare utilisation in the follow-up period. This finding reinforced previous studies on the high medical needs impact on hospitalisations and ED visits [4]. It is noteworthy for this Segment 2 to receive moderate programme utilisation during the follow-up period. Although the study did not look into the cause, it could be postulated from the high SOC visits that the segment had more unstable medical conditions.

Segment 3 as the reference group, had the lowest programme utilisation, healthcare utilisation and mortality in the follow-up period.

### Policy and research implications

Previous systematic review on predisposing, enabling and need characteristics of high cost patients included age, ethnicity, socioeconomic conditions, organisational factor (e.g., supply of health services and providers), medical conditions, health, and functional status. In these high-cost patients, inpatient services were most often reported as the main cost driver [4]. Furthermore, it reported that high-cost patients were more likely to die [4]. Our study reinforced the association between the characteristics of high users with healthcare utilisation and mortality.

Previous studies were mostly conducted in the United States and Canada, with a limited studies done in Asia [4–6]. It is plausible that there will be a diversity in patients’ characteristics and utilisation across different countries due to the difference in the epidemiological and health system factors. In addition, different countries might have different healthcare financing system, that could involve single payer or multi-payer framework.

Our study gave a more in-depth look on profiling the high users of inpatient services in Singapore. Moreover, it utilised the segmentation analysis approach to identify homogeneous population segments with similar characteristics and needs, to inform population health management and to allow for a tailored policy based on the local context.

This study reinforced the need to look at medical complexity, functional, and psychosocial needs as the distinguishing characteristics in segmenting high users of inpatient services into different population segments. In this study, Segment 1 with more functional dependency and psychosocial needs was found to have higher rate of programme utilisation, which included home visits done by either a doctor, nurse, physiotherapist, speech therapist, or occupational therapist. This suggests more needs for healthcare to support them with comprehensive assessment and care from the multi-disciplinary team. Segment 2 with more medical needs had the most need for hospital services. Studies reported that medically complex patients might benefit from more intensive medical models, including ambulatory intensive care units [4,28].

In this study of high users, the ones with the lowest medical, functional and psychosocial needs (Segment 3) comprised of almost half of the total population (44%) despite their relatively lesser demand on both programme and hospital services as compared to the Segments 1 and 2. Further profiling and differentiation of this group might aid in tailoring appropriate care to support them.

This study demonstrated that cluster analysis using administrative data could help identify patient segments with distinct healthcare characteristics and utilisation among patients with complex needs and who are high users of inpatient services. Majority of the studies on population segmentation included general population as the target population for segmentation, while others restricted to those with specific diseases or conditions [29]. This study further defined the target population at risk of adverse events i.e. complex high users of inpatient services, at risk of frequent hospital admission and mortality.

Most population segmentation systems adopt a healthcare utilisation risk-based rather than healthcare needs-based segmentation approach [2,30,31]. There is an increasing sentiment that segmentation based on healthcare utilisation risk is deemed inadequate to inform the development and allocation of healthcare services, as needs for specific healthcare services can occur due to few underlying phenomena, including the presence of morbidity risk, pain/discomfort, dysfunction, risk of mortality, patient’s subjective impression, or providers’ normative assessment [2]. Healthcare needs-based segmentation approach proposes a more comprehensive approach to individuals as it could provide indications for healthcare interventions that may lessen morbidity risk and address their needs [2]. Policy makers can optimise population health planning by applying healthcare needs-based population segmentation approach as an integral component in assessing population healthcare needs in relation to available services and resource allocation.

This study used CCI and ST as the segmentation variables, which are able to capture a comprehensive view of the individuals’ medical and psychosocial conditions, as a proxy of their needs. These variables not only covered the individuals’ risk of morbidity, but also their health-related behavior such as treatment compliance, and the wider determinants of health including their support system and coping response.

Most clustering algorithms depend on some assumptions in order to define the number of subgroups present in a data set. Similarly, the PAM approach required the number of clusters (*k*) to be pre-determined as input. As a consequence, the resulting clustering scheme requires some sort of evaluation as regards its validity and the goodness of clustering algorithm results [23]. Criteria to assess market segmentation have been widely adopted to evaluate the quality of population segmentation in healthcare context [13,32]. These include identifiability/interpretability (segments should be recognised and interpreted easily), substantiality (each segment should have sufficient size), stability (each segment should be relatively stable over time), and actionability/accessibility (each segments should be easily addressed and targeted with distinctive health intervention strategies) [13,32]. In this study, the segmentation outcomes demonstrated meaningful interpretation and clinical relevance as each segments were distinct in relation to its medical, functional and psychosocial needs pattern. In addition, each segments also differed in their rate and risk of future healthcare utilisation and mortality. These indicate a potential application for health service planning at population level.

There are a few study limitations. First, our study included older patients with high hospital service utilisation and complex chronic conditions, which may limit the generalisability of our findings to a more diverse and general population. Second, as the ST was not widely used and involved subjectivity from provider-rated assessment, its applicability to other populations requires further testing. Further studies might consider assessing the validity and reliability of the tool in different context and population. Third, we did not assess the segments performance against different data set. Hence, external validation and generalisability of the segmentation outcome requires further testing. Fourth, we did not include healthcare utilisation from other RHSs. However, majority of patients in Singapore utilise services within one regional health system [33]. Fifth, we did not conduct any analysis to compare healthcare utilisation and mortality in the excluded high users group, i.e., those discharged to institutional care or other community support services. Future studies might want to look into this population segment, to understand its characteristics, healthcare utilisation pattern, and risk of mortality. Lastly, this study is limited by the relatively short follow-up time. Future studies might consider having longer follow-up period to assess the stability of the segmentation outcome over time and also to track progression of each segment and transition between segments, thus allowing for the evaluation of targeted healthcare interventions and development of preventive healthcare services for each segment.

It is important to align the segmentation approach with the population segmentation objectives, as segmentation outcomes may differ with varied input variables and analytical approaches. Selection of variables for segmentation purposes requires iterative processes with contextual knowledge and expertise in data mining and clinical applicability.

### Conclusion

A cluster analysis approach in segmenting the population of complex high users of inpatient services using administrative data could identify segments with distinct characteristics and care needs. This study demonstrated the value in capturing a more comprehensive view on factors that influence the healthcare utilisation among complex high users of inpatient services. It provides evidence on healthcare needs-based segments that might potentially help policy makers in allocating resources efficiently by tailoring intervention to meet these needs. It also informs future research on the potential benefit of including data on psychosocial and nursing assessment to characterise complex high users of inpatient services.

## Supporting information

S1 Table Social Triage (ST) score. (DOCX)
