# Supplementary material for: Understanding the characteristics of high users of hospital services in Singapore and their associations with healthcare utilisation and mortality: A cluster analysis
Source: PLoS One. 2023 Jul 11;18(7):e0288441. doi: 10.1371/journal.pone.0288441 (PMC10335687; doi:10.1371/journal.pone.0288441)
Supplement: S1 Table — (DOCX) [file pone.0288441.s001.docx]

**S1 Table. Social Triage (ST) score**

| **Patient / family social support** | Existing caregiver unwilling / unable to provide support / no identified caregiver  (3 points) | Existing caregiver, express caregiver burden but open to options  (2 points) | Existing caregiver / formal or informal social support, willing to provide care  (1 point) |
| --- | --- | --- | --- |
| **Patient’s mental health** | Has dementia / depression / psychiatric / substance abuse / alcoholism / behaviour problem that affect care  (3 points) | Has dementia / depression / psychiatric / substance abuse / alcoholism / mild behaviour problem but not affecting care  (2 points) | Has good mental health, no issues  (1 point) |
| **Treatment compliance** | Poor compliance to treatment / care plans  (3 points) | Fair compliance to treatment / care plans  (2 points) | Good compliance to treatment / care plans  (1 point) |
| **Patient / family coping response** | Family / patient in shock, not accepting condition, grieving intensely / highly anxious  (3 points) | Family / patient accepting, still grieving / anxious but within control  (2 points) | Family / patient accepting and able to work on issues  (1 point) |

Total score: Low risk = 4-7 points; Moderate risk = 8-9 points; High risk = 10-12 points
